# Research on Leak Detection of Low-Pressure Gas Pipelines in Buildings Based on Improved Variational Mode Decomposition and Robust Kalman Filtering

**DOI:** 10.3390/s24144590

**Published:** 2024-07-15

**Authors:** Wenfeng Lin, Xinghao Tian

**Affiliations:** 1 School of Transportation Science and Engineering, Harbin Institute of Technology, Harbin 150001, China; 2 China Construction First Division Group Construction & Development Co., Ltd., Beijing 100102, China

**Keywords:** leak detection, low-pressure pipeline, VMD, robust Kalman filtering, scale space

## Abstract

Aiming at the complex characteristics of negative pressure waves in low-pressure pipelines inside of buildings, we proposed an estimation method of pressure fluctuation trends based on the robust Kalman filter and the improved VMD, which can be used for leakage detection. The reconstructed baseline signal can accurately describe the fluctuation trend of the negative pressure wave after the pressure drop, and quantitatively express the characteristic difference between the leakage condition and the gas usage condition. The robust Kalman filter was used to estimate the pressure fluctuations. The parameters of VMD were adaptively calculated based on the WAA and discrete scale space. The trend components contained in the IMFs were separated by a reconstruction based on the Fourier series. Based on the simulation signal, the method can accurately restore the trend component contained in the complex pressure signal. Based on the actual signals, the accuracy of small leakage detection is 96.7% and the accuracy of large leakage detection is 73.3%.

## 1. Introduction

Natural gas is a clean and low-carbon fossil energy which is of great significance to global environmental protection [1]. Pipelines are the main tool connecting natural gas resources with the market [2]. It should be noted that the leakage detection of low-pressure gas systems in buildings only depends on combustible gas alarms, which are passive, single, and weak. In the past four years, there have been approximately 3100 leaks in low-pressure gas pipelines in buildings in China [3]. Once the pipeline inside the building leaks, it is easy to accumulate, causing a series of secondary disasters such as combustion, explosions, and structural collapse [4,5]. The low-pressure gas pipeline system lacking an effective leak diagnosis method has become a fragile link in the lifeline of the city [6].

The research on the leak detection of low-pressure gas pipelines is almost blank. Related research has mainly focused on high-pressure gas pipelines and water networks outside of buildings [7,8]. In the development of pipeline risk identification and analysis technology, various methods have been proposed and applied with the advancement of sensors and digital signal processing technology [9,10]. By capturing the acoustic signal emitted by the pipeline leakage, the acoustic sensor can sensitively detect a sudden leakage [11]. By laying a distributed optical fiber with the pipe, it can detect the vibration of the pipe body caused by leakage [12]. The leaking steel pipe wall has defects, which can be judged by a magnetic sensor [13]. The pressure signal is one of the most easily obtained signals in pipeline monitoring, with rich features [14]. When the valve in the water network is suddenly closed, it will cause a relatively obvious water hammer pressure wave [15]. The presence of leaks in the pipeline will affect the frequency and phase of the water hammer to identify leaks [16,17]. Due to the nozzle structure of the burner, the pressure transient characteristics of the leakage condition and the gas usage condition in the low-pressure pipeline system are different. This was fully demonstrated in experimental tests and simulations without noise interference [3]. The negative pressure wave method is undoubtedly an effective leak detection method, but how it can be applied in the actual low-pressure pipelines full of noise interference has not been studied.

**Figure 1:**
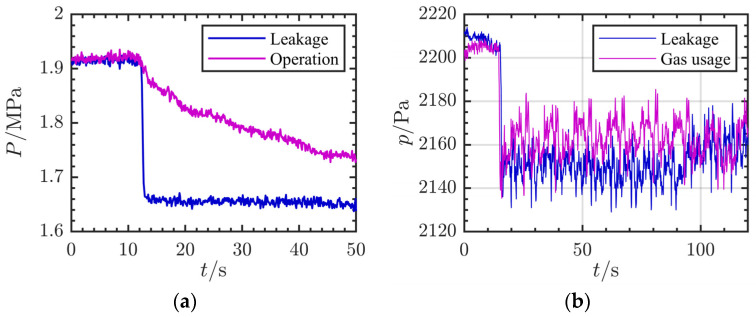
Schematic diagram of pressure fluctuations in different pipelines. (**a**) Pressure fluctuations in high-pressure pipelines outside buildings. (**b**) Pressure fluctuations in low-pressure pipelines inside buildings under actual conditions.

As shown in Figure 1, there is a significant difference in negative pressure waves between low-pressure and high-pressure pipelines. In low-pressure gas pipelines, the fluctuation characteristics of negative pressure waves under different conditions are severely obscured by noise. The lower the pressure, the more obvious the pressure wave reflected by the leakage [18]. But the noise caused by mechanical meters in low-pressure pipes is also obvious. When the flow rate is not zero, the pressure waveform of the low-pressure pipeline is full of large oscillations, which is obviously different from that of the high-pressure pipeline. This greatly increases the difficulty of detecting leakage pressure waves. The leak detection of low-pressure gas pipelines in buildings has the following particularities: (1) The gas supply pressure of the pipeline is generally about 2 kpa, which is almost suitable for civil gas burners all over the world [19]. (2) The length of the pipeline is generally within 5 m, so it is not necessary to locate the leakage. At the same time, the attenuation of the pressure wave can be ignored [3]. (3) The head end of the pipeline is connected with a membrane meter, which will produce periodic pressure fluctuations during the operation [20]. (4) With the frequent opening and closing of the terminal burner, there are a large number of negative pressure fluctuations, which is not conducive to the diagnosis of leakage [21]. Low-pressure gas pipelines are typically within 5 m in length and dispersed throughout the rooms. Hardware-dependent detection methods, including cable detection, radiological detection, magnetic leakage detection, ultrasonic detection, and fiber-optic detection, are too costly, too difficult to implement inside of the buildings, and cannot be detected in real time. Negative pressure waves mainly include the following two parts: a pressure drop and an oscillation fluctuation. The drop in pressure is usually instantaneous. The waveform of the pressure drop process in the time domain is similar to a straight line, and the slope angle is close to 90 degrees. The main difference between the different conditions is reflected in the trend of the oscillation fluctuation after the pressure drop [3]. The process of the fluctuation of pressure is disturbed by various noises. Therefore, the application of a negative pressure wave in indoor low-pressure pipeline leakage detection focuses on the accurate estimation of the pressure fluctuation trend. It is the basis for the identification of the leakage condition and gas usage condition. The noise brought by the membrane meter and the interference brought by the gas usage signal pose new challenges for the processing of pressure waves.

The pressure fluctuation signal in low-pressure gas pipelines is usually low-frequency, non-stationary, and multimodal. In recent years, the research hotspots of signal processing mainly include empirical mode decomposition (EMD) [22], variational mode decomposition (VMD) [23], wavelet analysis, empirical wavelet transforms, and particle filters. EMD decomposes signals according to time-scale features [24]. In addition to pressure waves, the continuous wavelet transform is also commonly used in leak detection based on acoustic signals [25]. A combination of EMD and the wavelet can be used to denoise the sensor signal and locate the leakage [26]. Due to the method of recursive filtering, EMD is prone to over-decomposition [27]. VMD is a new non-stationary signal processing method proposed in 2014; its decomposition process determines the center frequencies and bandwidths of the mode components after decomposition through an iterative search for an optimal solution relative to the variational model [23]. Compared with EMD, VMD reduces the problem of mode aliasing [28]. In the application process of VMD, the modal parameter K and the penalty parameter α need to be preset. In practical conditions, the prior knowledge of the signal is usually unknown. The deviation of the preset parameters will cause the decomposition of the signal to lose more real information [29]. Using a genetic algorithm to optimize the parameters of VMD can effectively extract the fault signal characteristics of the engine [30]. The particle swarm optimization algorithm can also be used to optimize the modal parameters and penalty parameters of VMD [31]. These optimization algorithms cannot avoid the disadvantages of a low efficiency and high computational power [32]. Based on the scale space, representation and binary clustering methods can be used to determine the modal parameter K of the bearing signal [33], which still requires the participation of prior knowledge. VMD is often used in conjunction with other filtering methods to denoise the signal [34]. Using the wavelet to denoise the signal in advance can improve the recognition accuracy of the ultrasonic guided wave by VMD [35]. Compared with other filtering methods, the Kalman filter considers the state information of past time and gives the optimal estimation, which has obvious advantages in the problem of trend estimation [36,37].

Considering the complex characteristics of the negative pressure wave signal in actual low-pressure pipelines, we proposed an estimation method of the pressure fluctuation trend based on the robust Kalman filter and improved VMD which can be used for leakage detection. The reconstructed baseline signal can accurately describe the fluctuation trend of the negative pressure wave after the pressure drop and quantitatively express the characteristic difference between the leakage condition and the gas usage condition.

The main theoretical research is organized as follows: Firstly, the robust Kalman filter is used to process the initial signal and remove the interference of background noise and observation noise. Secondly, the adaptive calculation method of the modal parameter and penalty parameter of VMD based on the discrete scale space and complex signal period extraction method is proposed. Finally, aiming at the boundary effect in the process of VMD decomposition, a signal reconstruction method based on the Fourier series is proposed. We established the simulation signals and collected the actual signals to verify the above theory. The method proposed in this paper provides theoretical support for the application of negative pressure waves in low-pressure pipelines in buildings, fills the gap of active leakage detection in low-pressure gas pipelines in buildings, and can effectively solve the shortcomings of the low leakage detection accuracy of traditional threshold detection methods.

The structure of this paper is organized as follows: Section 2 introduces the principles of the robust Kalman filter and the improved VMD. Section 3 introduces the implementation process of the above methods for leakage detection. In Section 4, the simulation signal model is established, and the above methods are verified based on the simulation signals. In Section 5, the actual data acquisition system is built, and the above methods are verified based on the actual signals. Section 6 summarizes the relevant conclusions.

## 2. Methodology

### 2.1. Robust Kalman Filter

Considering the gross error and random noise in the observation data, we used the robust Kalman filter based on M-estimation. The filtering equation is generally described by a linearized discrete model, which can be expressed as follows:

(1) $$X_{k}=F_{k,k-1}X_{k-1}+W_{k}$$

(2) $$Y_{k}=H_{k}X_{k}+V_{k}$$

where $X_{k}$ is the state vector; $Y_{k}$ is the observation vector; $W_{k}$ is the systematic error vector; $V_{k}$ is the observation error vector; $F_{k,k-1}$ is the state transition matrix; $H_{k}$ is the observation coefficient matrix.

Through the historical state, we can recursively obtain the estimated state ${\hat{X}}_{k}$ and its covariance matrix $P_{{\hat{X}}_{k}}$ at the current time. The recursive process can be expressed as follows [38]:

(3) $${\bar{X}}_{k}=F_{k,k-1}{\hat{X}}_{k-1}$$

(4) $$P_{{\bar{X}}_{k}}=F_{k,k-1}P_{{\hat{X}}_{k-1}}F_{k,k-1}^{T}+Q_{k}$$

(5) $$K_{k}=P_{{\bar{X}}_{k}}H_{k}^{T}{\left(H_{k}P_{{\bar{X}}_{k}}H_{k}^{T}+R_{k}\right)}^{-1}$$

(6) $${\hat{X}}_{k-1}={\bar{X}}_{k}+K_{k}(Y_{k}-H_{k}{\bar{X}}_{k})$$

(7) $$P_{{\hat{X}}_{k}}=P_{{\bar{X}}_{k}}(1-K_{k}H_{k})$$

where ${\bar{X}}_{k}$ and $P_{{\bar{X}}_{k}}$ are the prediction state of the current time and its covariance matrix; $Q_{k}$ is the system error matrix; $R_{k}$ is the observation error matrix; $K_{k}$ is the Kalman gain matrix.

Based on the principle of M-estimation, we introduced the equivalent weight matrix ${\bar{P}}_{k}$ of the observation vector to calculate the equivalent Kalman gain matrix ${\bar{K}}_{k}$, which can be expressed as follows:

(8) $${\bar{K}}_{k}=P_{{\bar{X}}_{k}}H_{k}^{T}{\left(H_{k}P_{{\bar{X}}_{k}}H_{k}^{T}+{\bar{P}}_{k}\right)}^{-1}$$

In the process of the robust estimation, the equivalent gain matrix ${\bar{K}}_{k}$ replaces the original gain matrix $K_{k}$. In this paper, the method of updating the observation value of the equivalent weight matrix ${\bar{P}}_{k}$ depends on the IGG III function, and more details can be found in [39].

### 2.2. Variational Mode Decomposition

VMD decomposes signals by finding the optimal solution to a constrained variational decomposition problem. It follows the concept of the intrinsic mode function (IMF) in EMD. In theory, all signals f(t) can be decomposed by VMD into *K* mode functions $u_{k}(t)$ (k=1,2,3,…,K) centered at $\omega_{k}(t)$ [33]. Combined with the Hilbert transform, VMD seeks to minimize the sum of the bandwidths of the *K* modes. The constrained variational model can be simply expressed as follows [40]:

(9) $$u_{k}(t)=A_{k}(t)\times \cos(\varphi_{k}(t))$$

(10) $$\underset{\left\{u_{k},\omega_{k}\right\}}{\min}\left\{\sum_{k}{\left\|\partial_{t}[(\delta (t)+\frac{j}{\pi t})\times u_{k}(t)]e^{-j\omega_{k}t}\right\|}_{2}^{2}\right\}$$

(11) $$s.t.\sum_{k}u_{k}=f$$

where δ(t) is the pulse signal; $\varphi_{k}(t)$ is a non-decreasing phase function.

Based on the above model, the Lagrange multiplication operator is introduced into the calculation, which can transform the constrained variational problem into an unconstrained variational problem. The mathematical expression for the augmented Lagrange function is as follows [34]:

(12) $$\begin{array}{c} L\left(\left\{u_{k}\right\},\left\{\omega_{k}\right\},\lambda \right)=\alpha {\sum_{k}\left\|\partial_{t}\left[\left(\delta (t)+\frac{j}{\pi t}\right)u_{k}(t)\right]e^{-j\omega_{k}t}\right\|}_{2}^{2}\\ +{\left\|f(t)-\sum_{k}u_{k}(t)\right\|}_{2}^{2}+\langle \lambda (t),f(t)-\sum_{k}u_{k}(t)\rangle \end{array}$$

where α is the penalty parameter and λ is the Lagrange multiplier.

In order to convert the above model to the frequency domain for calculation, it is necessary to introduce the Fourier equidistant variation based on Parseval theory and the Plancherel theorem. The update process of the center frequency of each IMF can be expressed as follows [34]:

(13) $${\hat{u}}_{k}^{n+1}(\omega )=\left(\hat{f}(\omega )-\sum_{i\neq k}{\hat{u}}_{i}(\omega )+\frac{\hat{\lambda }(\omega )}{2}\right)\frac{1}{1+2\alpha {\left(\omega -\omega_{k}\right)}^{2}}$$

where ${\hat{u}}_{k}^{n+1}(\omega )$, $\hat{f}(\omega )$, and $\hat{\lambda }(\omega )$ represent the Fourier transformations of $u_{k}^{n+1}$, $f\left(t\right)$, and $\lambda \left(t\right)$. ${\hat{u}}_{k}^{n+1}(\omega )$ can be considered as the Wiener filter of $\hat{f}(\omega )-\sum_{i\neq k}{\hat{u}}_{i}(\omega )$. $\omega_{k}^{n+1}$ is the gravity center of the power spectrum of the current mode function and can be expressed as follows:

(14) $$\omega_{k}^{n+1}=\frac{\int_{0}^{\infty }\omega {\left|{\hat{u}}_{k}^{n+1}\left(\omega \right)\right|}^{2}d\omega }{\int_{0}^{\infty }{\left|{\hat{u}}_{k}^{n+1}\left(\omega \right)\right|}^{2}d\omega }$$

The cyclic process of VMD takes the ratio of the difference before and after each IMF update to the ${\hat{u}}_{k}^{n}$ value before the update as the control error. It can be expressed as follows [33]:

(15) $$\frac{\sum_{k}{\left\|{\hat{u}}_{k}^{n+1}-{\hat{u}}_{k}^{n+1}\right\|}_{2}^{2}}{{\left\|{\hat{u}}_{k}^{n}\right\|}_{2}^{2}}<\varepsilon$$

where ε is the convergence tolerance.

### 2.3. Adaptive Calculation of Modal Parameter K Based on Improved Scale-Space Theory

The modal parameter *K* determines the number of IMFs decomposed by VMD, which is very important for accurately extracting the trend of pressure fluctuations. The purpose of processing the pressure signal of the low-pressure pipeline is to extract the baseline of the negative pressure wave in the pressure oscillation section, which includes the trend of the transient pressure fluctuation and the characteristics of the leakage condition [3]. The frequency of periodic noise caused by membrane meters is relatively low, and it is difficult to easily separate from the fluctuating baseline [20]. In this section, we introduce scale-space theory to process the frequency spectrum of the pressure signal. A complex pressure signal period calculation method based on morphological filtering is proposed, which can accurately detect the frequency of the periodic noise caused by the membrane meter. The modal parameter *K* can be calculated adaptively through the scale-space representation of the frequency spectrum and the periodic calculation of the membrane meter signal.

#### 2.3.1. Scale-Space Representation of the Frequency Spectrum

The scale-space representation of discrete signals was proposed by Lindeberg in 1990 [41]. The theory characterizes the discrete signal from detail feature to contour feature by a set of multi-resolution methods. In 2014, Gilles proposed to treat the spectrum of the signal as a histogram, so that the effective frequency band in the spectrum can be identified by using the scale-space theory [42].

Assuming that there is a signal f(t) in the interval [0, $t_{\max}$], the spatial scale representation of the signal f(t) can be defined as follows [43]:

(16) $$L\left(m,\sigma \right)=\sum_{n=-\infty }^{+\infty }f\left(m-n\right)g\left(n,\sigma \right)\approx \sum_{n=-M}^{+M}f\left(m-n\right)g\left(n,\sigma \right)$$

where $g\left(n,\sigma \right)$ is the kernel function for the scale-space computation; *M* is the length of the filter when it is actually applied; σ is the scale parameter of the kernel function. Generally, g uses a Gaussian kernel function, which can be expressed as follows:

(17) $$g\left(n,\sigma \right)=\frac{1}{\sqrt{2\pi \sigma }}e^{-\frac{n^{2}}{2\sigma }}$$

When *M* is large enough, the approximation error ($2\int_{M}^{\infty }g\left(n,\sigma \right)dn$) of the finite impulse response filter can be ignored. With the update of the scale σ, *M* is generally taken as follows:

(18) $$M=C\sqrt{\sigma }+1$$

(19) $$\sqrt{\sigma }=s\sqrt{\sigma_{0}},s\in [1,2t_{\max}]$$

where *C* is the empirical coefficient, generally taken as 6; $\sigma_{0}$ is the initial scale, generally taken as 0.5; s is the number of iterations of the scale parameter σ, up to twice the signal length $x_{\max}$.

The essence of scale-space representation is to convolute signals with kernel functions of different scales. The larger the scale parameter of the kernel function, the higher the smoothness of the signal. According to Lindeberg’s theory, the number of local minima of the function $L\left(m,\sigma \right)$ decreases as the scale parameter σ increases [41]. It needs to be clear that, in the process of updating the scale parameter, the function $L\left(m,\sigma \right)$ will not generate a new local minimum value.

The number of local minima in the initial scale is recorded as $N_{0}$. Each minimum corresponds to a scale-space curve $CL_{i}(i=1,2,3,\ldots N_{0})$, which represents the life of the local minimum in the process of scale transformation. The length $L_{i}$ of the curve $CL_{i}$ can be approximately defined as follows:

(20) $$L_{i}=\max\left\{\frac{s}{\mathrm{the}\;i\mathrm{th}\;\mathrm{minimum}\;\mathrm{exists}}\right\}$$

#### 2.3.2. Period Calculation of Low-Frequency Noise in Complex Signals

Gilles believes that the local minimum value in the signal spectrum is an important basis for dividing the effective frequency band [42]. The threshold of the scale-space curve $CL_{i}$ determines the number of valid frequency band divisions. In the actual low-pressure pipeline pressure signal, the periodic noise of the membrane meter interferes the most with the frequency spectrum division. Based on morphological filtering, we proposed a period calculation method which can effectively extract the period and dominant frequency of the low-frequency noise in complex signals. The extracted dominant frequency of the periodic noise can be used as a basis for the adaptive determination of the threshold of spatial scale curves.

Morphological filtering contains a variety of structures and operation types [44]. Through the white hat transformation (WTH), the periodic information of the complex pressure signal can be accurately extracted by the autocorrelation function (ACF) and the average amplitude difference function (AMDF). The WTH performs a morphological opening operation on the signal, and then filters out the original signal. It can highlight the peak characteristics of the signal. Assuming a discrete signal f(n),n=1,2,3…N, the transform process can be expressed as follows [45]:

(21) (f⊕z)(n)=max[f(n−m)+z(m)]

(22) (fΘz)(n)=min[f(n−m)+z(m)]

(23) (f∘g)(n)=(fΘg−⊕g)(n)

(24) WTH(n)=f(n)−(f∘g)(n)

where z(m) is the structural element; f⊕z is the expansion operation, which can increase the number of local minimum values of the signal; fΘz is the corrosion operation, which can reduce the number of local maxima of the signal; f∘g is the opening operation, which can suppress the peak of the signal to achieve the smoothing effect.

On the basis of the WTH, we introduced the ACF and AMDF for further signal processing. The ACF and AMDF are only suitable for simple signals with obvious periods [46,47]. The calculation process can be expressed as follows:

(25) $$ACF(n)=\sum_{m=1}^{N-(n-1)}f(n)f(n+m),n=1,2,3,\ldots ,N$$

(26) $$AMDF(n)=\sum_{m=1}^{N-(n-1)}\left|f(n)-f(n+m)\right|,n=1,2,3,\ldots ,N$$

The ACF and AMDF have been applied in the period detection of some audio signals and vibration signals [48]. Considering the characteristics of the pressure signal of the low-pressure pipeline, we proposed the method of the combined application of the WTH, ACF, and AMDF (WAA), which is defined as follows:

(27) $$WAA(n)=\frac{\sum_{m=1}^{N-(n-1)}WTH(n)WTH(n+m)}{\sum_{m=1}^{N-(n-1)}\left|WTH(n)-WTH(n+m)\right|},n=1,2,3,\ldots ,N$$

The local maximum interval in the WAA waveform represents the period of the noise signal of the membrane meter, and the reciprocal of the period is considered to be the dominant frequency. The WAA avoids the interference of various peaks in the signal spectrum and directly locates the frequency band of the periodic noise.

The membrane meter is a mechanical structure. A complete cycle of outlet pressure fluctuation represents the gas passing through a fixed volume. When the periodic fluctuation of the membrane meter is too dense, it means that a large flow leakage occurs in the pipeline. Therefore, another function of the WAA is to judge the occurrence of a large leakage as a simple calculation method.

#### 2.3.3. Adaptive Method of Scale-Space Threshold Based on WAA

Through the WAA, the dominant frequency of periodic noise caused by the membrane meter can be obtained. In the spectrum of the signal, there may be several local minima between the membrane meter frequency and the baseline frequency. Accordingly, there may be several curves between the two frequencies in the scale space of the spectrum. We have identified the longest scale-space curve length below the periodic noise frequency as the scale-space curve threshold. It can be ensured that the periodic noise component can be successfully separated from the pressure fluctuation baseline component adaptively. The number of spatial curves whose lengths are greater than the threshold can be used as the modal parameter *K* of VMD.

### 2.4. Adaptive Calculation Model of Penalty Parameter

The penalty parameter α has a certain influence on the convergence speed and decomposition results of VMD. If its value is too small, the wider the frequency band of each IMF, the more susceptible it is to spectrum aliasing. In the pressure signal of low-pressure pipelines, the baseline frequency representing the trend is very close to the periodic noise frequency of the membrane meter. When the modal parameter *K* is relatively accurate, the penalty parameter needs to be set high enough to ensure that the periodic noise can be separated and filtered. By combining the information of the divided frequency band with the sampling frequency, Li constructed the value model of the penalty factor [49]. Considering that the penalty factor is inversely proportional to the bandwidth of the IMF [50], we proposed an improved penalty parameter calculation method. Based on the dominant frequency of periodic noise and the sampling frequency, α can be calculated adaptively as follows:

(28) $$\alpha =\left(\frac{1}{1+e^{\log10\frac{2f_{p}}{f_{s}}}}-0.5\right)e^{2f_{s}}$$

where $f_{p}$ is the dominant frequency of the periodic noise detected by the WAA; $f_{s}$ is the sampling frequency. The larger the dominant frequency $f_{p}$ of the periodic noise, the easier it is to separate from the trend baseline, and the smaller the penalty parameter α can be set. Therefore, $f_{p}$ is negatively correlated with the penalty parameter α.

### 2.5. Adaptive Signal Reconstruction Method Based on Fourier Series

According to the above theory, the modal function $u_{1}(t)$ with the lowest frequency obtained by VMD decomposition basically represents the trend of pressure transient oscillation. However, some baseline trends are also included in the modal functions $u_{k}(t)$ ($k=2,3,\ldots ,K_{1}$), representing low-frequency noise, due to the boundary effect of the actual signal. Considering the frequency characteristics of the noise, we proposed an adaptive signal reconstruction method based on the Fourier series.

The periodic fluctuation characteristics of noise are obvious relative to the baseline fluctuations. The Fourier series can fit the periodic signals very well. As the order increases, the Fourier series fits the signal from global to local. If the order is unchanged, the description scale of the Fourier series is essentially unchanged for the same signal.

The trend components are the local details of each low-frequency noise mode due to the boundary effects. It can generally be considered that the overall characteristics of noise do not change with time. We can use the Fourier series to fit the low-frequency noise modes that do not contain trend components. Through error control, the order of the Fourier series that can accurately describe the overall characteristics of the noise that is obtained. Fitting the complete noise mode with the same order, the Fourier series will still give priority to accurately describing the overall characteristics of the noise over the local details. Therefore, it can be considered that the reconstructed low-frequency noise mode basically contains no trend components.

As mentioned in Section 1, we only care about the pressure fluctuation process of the negative pressure wave after the pressure drop is completed. Therefore, the onset of f(t) is usually the lowest point of the pressure drop $t_{0}$, that is, $t\in \left[t_{0},t_{0}+t_{\max}\right]$. Generally, the duration of the boundary effect generally does not exceed 20 S [3]. The low-frequency noise modes ${u^{\prime }}_{k}(t)$ without trend components can be expressed as follows:

(29) $${u^{\prime }}_{k}(t)=u_{k}(t),k\in \left[2,K_{1}\right],t\in \left[t_{0}+20,t_{0}+t_{\max}\right]$$

(1) ${u^{\prime }}_{k}(t)$ can be accurately described by Fourier fitting. Gradually increasing the order $I_{k}$ of the Fourier series makes the fitting error gradually smaller.

(2) The fitting error of ${u^{\prime }}_{k}(t)$ is the mean square error $MSE_{k}$. The error threshold $\varepsilon_{k}$ is generally set to 2% of the variance of ${u^{\prime }}_{k}(t)$.

(30) $$\begin{array}{c} {u^{\prime }}_{k}(t)≃{u^{\prime \prime }}_{k}(t)=\frac{a_{0}}{2}+\sum_{i=1}^{I_{k}}a_{i}\cos it+b_{i}\sin it\\ k\in \left[2,K_{1}\right],t\in \left[t_{0}+20,t_{0}+t_{\max}\right] \end{array}$$

(31) $$MSE_{k}=\frac{1}{t_{\max}-20}\sum_{t=t_{0}+20}^{t_{\max}}{\left({u^{\prime \prime }}_{k}(t)-{u^{\prime }}_{k}(t)\right)}^{2}\leq \varepsilon_{k}$$

(3) The complete noise mode $u_{k}(t)$ is reconstructed by a Fourier series with the same order $I_{k}$ to obtain the reconstructed signal ${\hat{u}}_{k}(t)$.

(32) $$\begin{array}{c} u_{k}(t)≃{\hat{u}}_{k}(t)=\frac{a_{0}}{2}+\sum_{i=1}^{I_{k}}a_{i}\cos it+b_{i}\sin it\\ k\in \left[2,K_{1}\right],t\in \left[t_{0},t_{0}+t_{\max}\right] \end{array}$$

(4) The accurate baseline trend $f_{b}(t)$ can be calculated as follows:

(33) $$f_{b}(t)=\sum_{k=2}^{K_{1}}u_{k}(t)-\sum_{k=2}^{K_{1}}{\hat{u}}_{k}(t)$$

Trend components are included in the low-frequency IMFs within 0.5 Hz. Generally, the low-frequency part of the actual pressure signal can be divided into three effective frequency bands at most. When there are fewer than three low-frequency bands containing trend components, the multi-calculated high-frequency bands have little impact on the final reconstruction results.

## 3. Implementation Process for Leak Detection

Through the proposed methods, we have established a complete set of processing flows for indoor low-pressure pipeline pressure signals. The improved VMD method based on the robust Kalman filter (IKVMD) can effectively extract the fluctuation trend after the pressure drops. Combined with the trend feature indicators mentioned in [3], accurate indoor gas system leak detection can be achieved. The detailed steps of applying the proposed method to leak detection are as follows:

(1) The pressure signals are collected by the sensor at the starting point of the low-pressure pipeline system.
(2) The collected pressure signals are denoised by the robust Kalman filter, so as to improve the accuracy of the VMD decomposition.
(3) The periodic detection is carried out through the WAA to calculate the dominant frequency of the low-frequency periodic noise $f_{p}$ in complex signals.
(4) The spectra of the signals are characterized in discrete scale space, and the threshold is adaptively determined to obtain the modal parameter *K* of VMD.
(5) According to the dominant frequency of the periodic noise $f_{p}$ and the sampling frequency $f_{s}$, the penalty parameter α of VMD is calculated by Equation (28).
(6) Based on the parameters mentioned above, VMD is used to decompose the denoised signal.
(7) The decomposed signal is reconstructed based on the Fourier series to obtain the baseline representing the pressure fluctuation trend.
(8) The trend feature indicators [3] of the baseline are calculated and the conditions represented by the pressure fluctuation trend are identified.

## 4. Simulation Study

In order to verify the effectiveness of the proposed IKVMD method, we first need to use the simulation signals for discussion. The simulation signals can well evaluate the effect of signal processing.

### 4.1. Simulation Signals

When the pressure drop occurs, the pressure signal components become complex and can be divided into the following four categories: (1) the baseline signal representing the pressure fluctuation trend; (2) the periodic noise caused by the membrane meter; (3) the background noise of the system; (4) the measurement noise of the sensor. The components of the signal are the same in the gas usage condition and leakage condition. The difference lies in the size of the pressure drop and the trend of the pressure fluctuation after the pressure drop.

**Figure 2:**
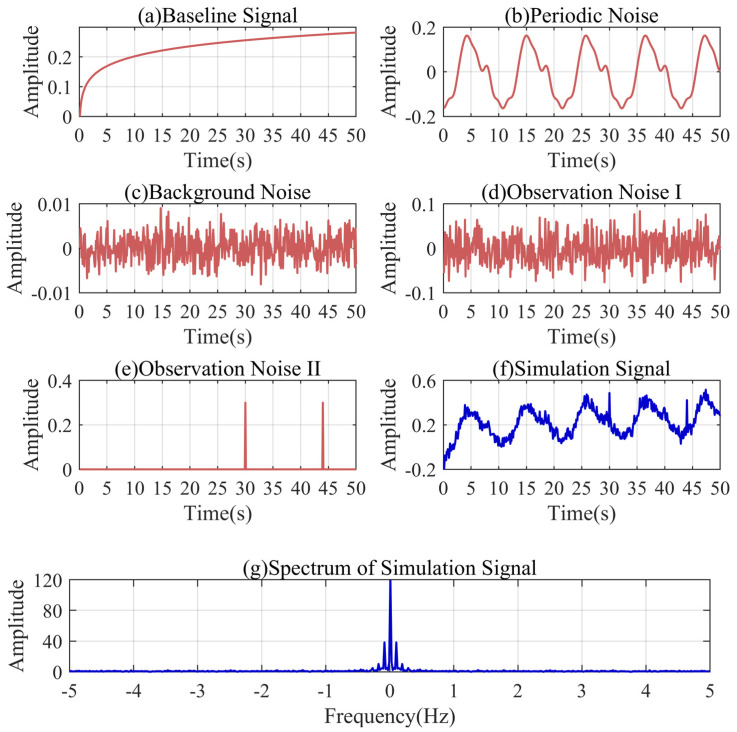
Composition and spectrum of the simulation signal.

Based on the above discussion, one type of simulation signal of a similar condition is sufficient to illustrate the accuracy of the IKVMD for the pressure wave trend extraction. Therefore, we simulated the fluctuation process after the pressure drop in the actual leakage process of the low-pressure pipeline for discussion. Figure 2 shows the composition and spectrum of the simulation signal. As shown in Figure 2a, the baseline signal in the simulation signal can be represented as follows:

(34) $$f_{b}(t)=1.126t^{0.039}-1.025,\;t\in [0.1,50]$$

The models of noise signals are relatively simple. The periodic noise is a six-stage Fourier series similar to the actual signal. The background noise is Gaussian white noise with a variance of one hundred thousandth. The observation noise includes Gaussian white noise with variance of one thousandth and the gross error. As shown in Figure 2f, the simulation signal can be obtained through the superposition of the baseline signal and noise signals. The fluctuation characteristics of the simulation signal are basically consistent with the actual pressure signal characteristics of the low-pressure pipeline. Combined with Figure 2g, it can be found that there are multiple peaks in the low-frequency region of the simulation signal. The frequency of the periodic noise is close to that of the baseline signal.

### 4.2. Simulation Signal Denoising Based on Robust Kalman Filter

**Figure 3:**
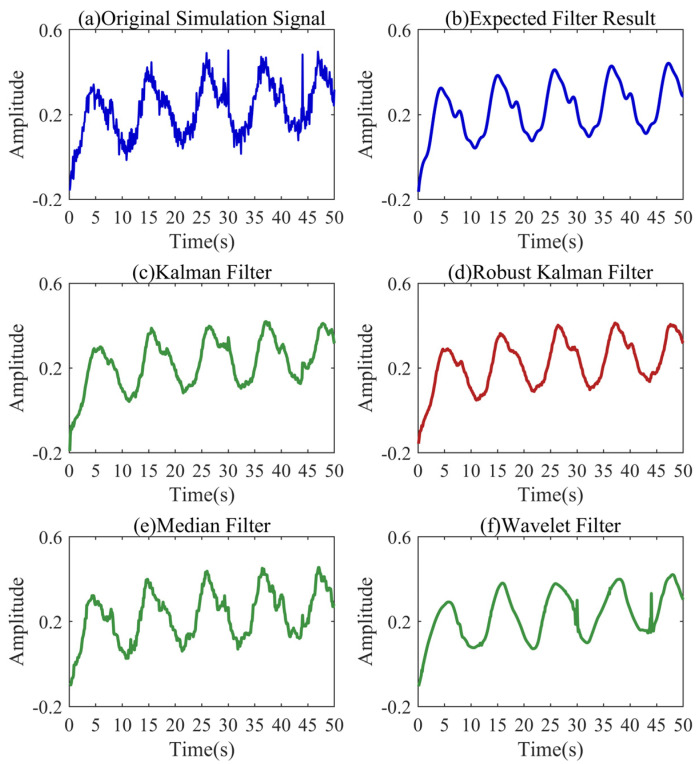
Denoising results of the filters based on the simulation signal.

As shown in Figure 3, we explored the performance of various filters for denoising based on the simulation signal. Figure 3a shows the signal before filtering, and Figure 3b shows the expected filtering result. The systematic and observed error parameters of the Kalman filter are consistent with the simulated signal. As shown in Figure 3c, it restores the expected waveform well, but the coarse errors at the positions of 30 s and 44 s cause a great disturbance to the Kalman filter. The median filter uses a window of a length of 0.5 s for filtering. As shown in Figure 3e, it restores the expected waveform in general, and weakens the initial fluctuation of the signal. In addition, the gross errors are not handled well. It should be pointed out that the initial fluctuation of the signal is very critical in the trend of the signal and is not expected to be weakened. The wavelet filter uses the basis function sym5 to perform the five-layer soft threshold denoising. As shown in Figure 3f, the wavelet filter has a low degree of restoration of the expected waveform, and it is difficult to eliminate the influence of gross errors. As shown in Figure 3d, the robust Kalman filter restores the expected waveform the best and basically eliminates the influence of gross errors. The robust Kalman filtering has obvious advantages in denoising complex pressure signals in low-pressure pipelines.

### 4.3. Simulation Signal Period Extraction Based on WAA

**Figure 4:**
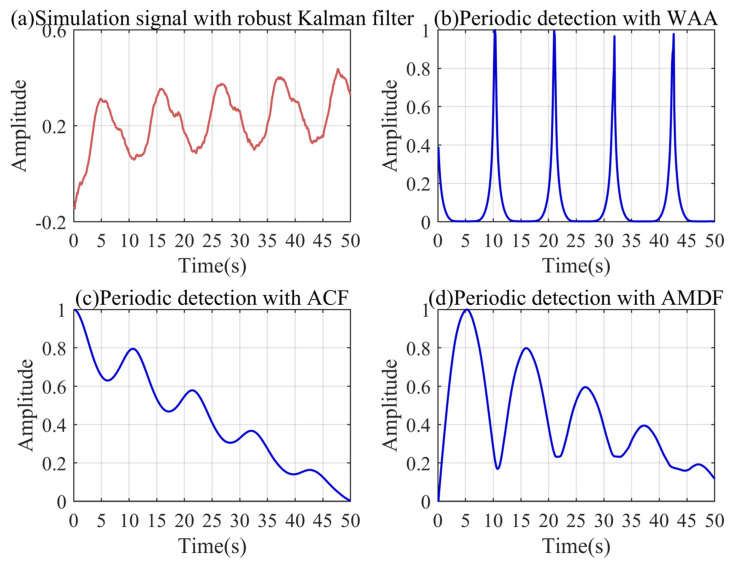
Period extraction results based on the simulation signal.

Before decomposing the signal through VMD, we need to extract the period of the signal by the WAA to determine the threshold of the discrete scale-space curve. Figure 4 shows the period extraction results based on the simulation signal. Figure 4a is the simulation signal after the robust Kalman filter, which is used for the period extraction. It can be found that the peak amplitudes of the ACF and AMDF decrease gradually. The peak structures of the two functions also gradually become blurred. In comparison, the peak value of the WAA is convex and the amplitude is stable. The sharp peak of the WAA accurately corresponds to the end of each signal period in the simulated signal. This is mainly because the peak structure of periodic noise is obvious, and the WTH can highlight the peak characteristics of the signal. Because the peak characteristics of the periodic noise in the actual signal are more prominent, the advantage of the WAA will be more obvious in the actual signal, which is more fully proven in Section 5.3.

### 4.4. Adaptive Spectrum Partition Based on Discrete Scale Space

**Figure 5:**
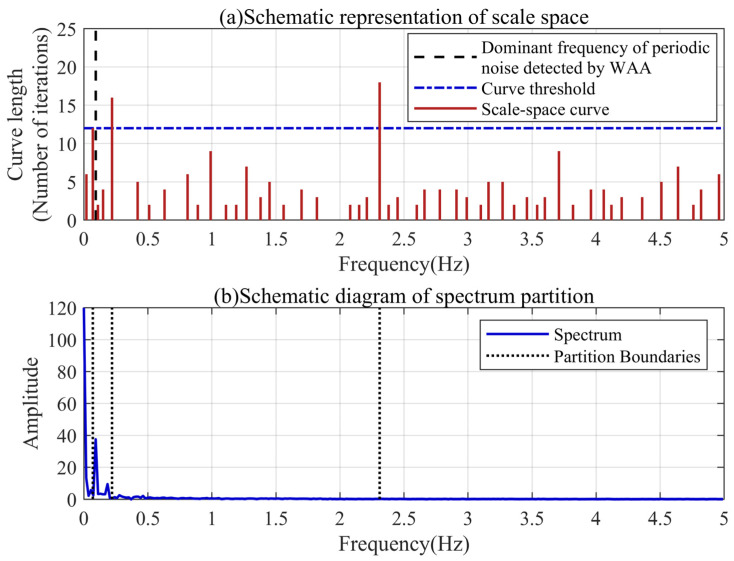
Spectrum partition results based on discrete scale space.

The modal parameter *K* represents the number of effective frequency bands in the spectrum. Based on the dominant frequency of the periodic noise detected by the WAA, we can adaptively segment the spectrum of the filtered signal through the scale space. As shown in Figure 5, the number of local minima in the signal spectrum is decreasing with the continuous iteration of the scale space. The length of the scale-space curve represents the number of iterations of the local minimum that exists in the signal spectrum. The longer the curve, the easier it is to separate the frequency bands before and after the local minimum. Dividing the frequency bands according to the different lengths of the scale-space curves can reduce the possibility of frequency band aliasing.

It can be found that there are two curves in the frequency region smaller than the periodic noise extracted based on the WAA, which means that there are two local minima. It also shows that there are multiple spectral peaks in the low-frequency region, and the WAA can simply locate the dominant frequency of the periodic noise. The longest of the two curves corresponds to the frequency at which the periodic noise and baseline trend are most easily separated. Its length can be used as a threshold for the scale-space curve. There are three curves in the scale space that are not less than this threshold, and the spectrum can be divided into four parts, which means that the modal parameter *K* can be set to four.

### 4.5. Improved VMD Decomposition of Simulation Signal

**Figure 6:**
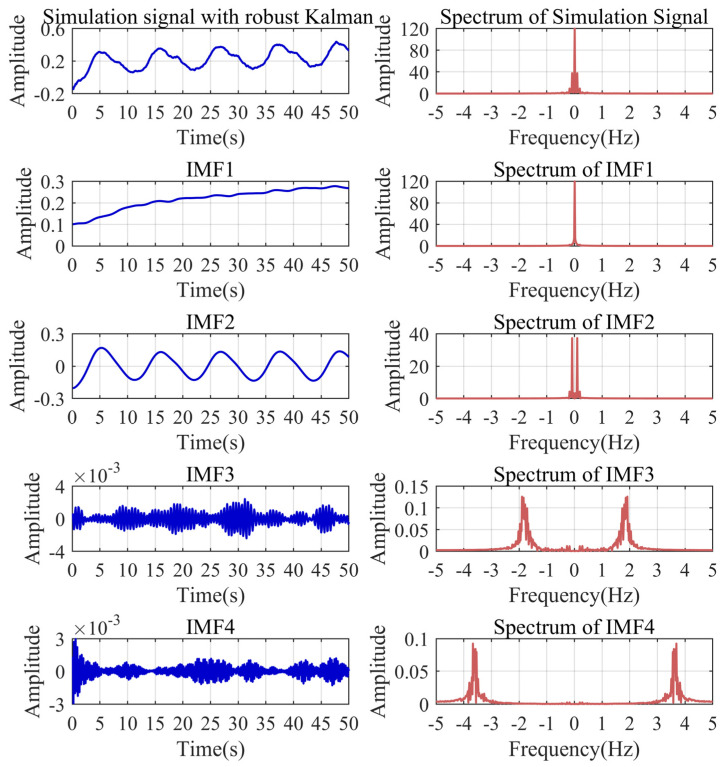
VMD decomposition results of the simulation signal.

Through the calculation of the WAA and scale space, the modal parameter *K* and penalty parameter α can be adaptively calculated as four and 7685. On this basis, we performed VMD decomposition on the simulation signal after the robust Kalman filtering. As shown in Figure 6, the signal is decomposed into four IMFs. The frequency boundaries of the IMFs are basically the same as the preset boundaries of the scale space. There is almost no modal aliasing among the IMFs. The baseline trend and periodic noise are successfully separated without increasing the number of modes. This illustrates the effectiveness of the scale space for spectral partitioning. It should be noted that the initial part in the IMF2 still contains part of the baseline trend, limited by the boundary effect of the signal. Therefore, we need to utilize the reconstruction of the signal to accomplish a complete separation of the baseline trends.

### 4.6. Reconstruction Results of Simulation Signal Based on Fourier Series

**Figure 7:**
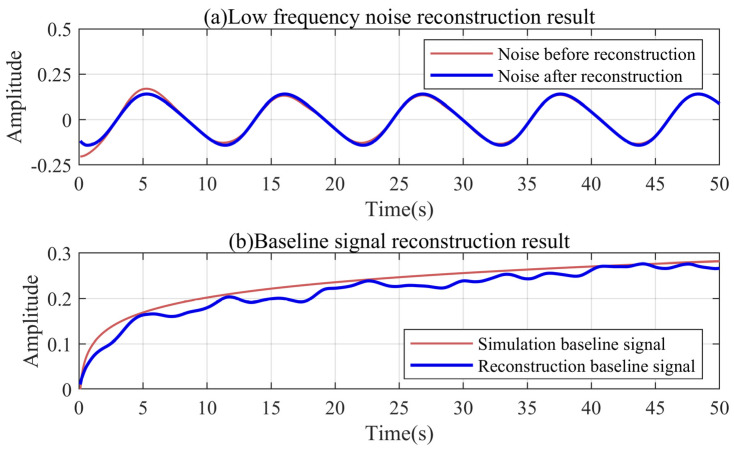
Schematic diagram of the simulation signal reconstruction process.

The Fourier series can accurately describe the overall characteristics of the noise. In the decomposition result of the simulation signal, the low-frequency noise of less than 0.5 Hz is only the IMF2. In order to illustrate the filtering effect of the Fourier series on the boundary effect, we set the number of low-frequency modes $K_{1}$ to two. By performing multiple Fourier series fitting on the IMF2 without the trend component, the minimum order is three under the premise of controlling the error. The complete IMF2 is then reconstructed with a third-order Fourier series. As shown in Figure 7a, the third-order Fourier series still accurately describes the IMF2 without the trend component. Correspondingly, the trend component located at the boundary is ignored in the reconstruction process. This is because the third-order Fourier series will preferentially fit the global features of the IMF2 rather than local details.

We reconstructed the baseline signal with Equation (33). As shown in Figure 7b, the reconstructed baseline signal is basically the same as the simulation signal. The error at the starting point of the reconstructed signal is 0.0104. The simulation signal can be viewed as the upper envelope of the reconstructed signal. Small local errors are unavoidable and can be ignored. After the robust Kalman filtering and the improved VMD decomposition, the reconstructed baseline signal relatively accurately expresses the trend of the complex signal fluctuations.

## 5. Leak Detection Based on Actual Signals

### 5.1. Actual Signals

**Figure 8:**
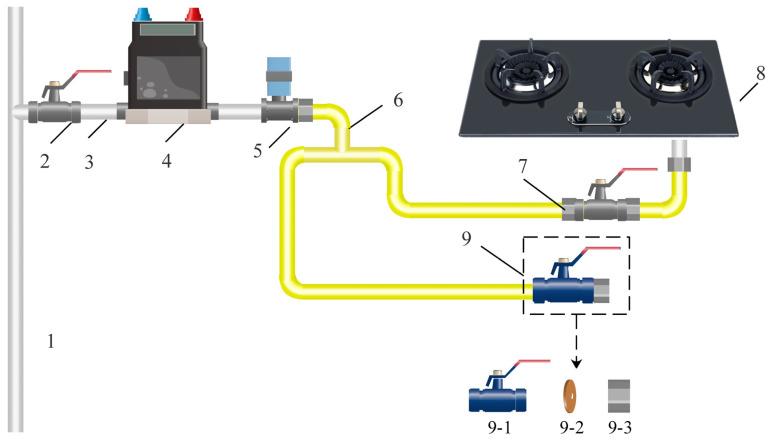
Structural diagram of the indoor pipeline system with a leakage simulation branch pipe. 1—vertical pipeline of the building internal combustion gas; 2—main valve; 3—iron tube; 4—flow meter; 5—pressure monitoring equipment; 6—connecting hose; 7—manual valve; 8—gas stoves, 9—leakage simulation device; 9-1—manual valve; 9-2—copper gasket; 9-3—fixing nut.

In order to obtain the actual signals to verify the performance of the IKVMD for leak detection, we selected the actual indoor pipeline systems of three households for monitoring. As shown in Figure 8, the selected indoor pipeline systems are representative in structure. The pressure monitoring devices are installed on the branch of the vertical gas pipes, with a distance of about 0.3 m. The lengths of the connecting pipes between the pressure monitoring device and the stove are 2–3 m. Limited by the area of the kitchen and the position of the vertical gas pipe, the distance between most gas stoves and the vertical pipes is within 3 m. The connecting hoses are rubber hoses with a diameter of 10 mm. The gas supply pressure of the piping systems is 2000–2300 Pa. The types of the gas stoves are the JZT-Q230(12T), DS325S(12T), and KA022B. The rated heat loads of the gas stoves are 4.1–4.5 KW, which are the common heat load value of household gas stoves. The nozzle diameters of the natural gas stoves are all 1.2 mm.

In addition, we added a leakage simulation branch to the indoor pipeline. As shown in Figure 8, the leakage simulation branch is mainly composed of a leakage simulation device and connecting hose. The distance between the leakage simulation device and the pressure monitoring device was set to 2.3 m. The core of the leakage simulation device is a gasket with a central circular hole. We simulated the occurrence of leakage in the pipeline by controlling the opening of the end manual valve. There are two kinds of diameters of round holes, 0.4 mm and 1.2 mm, which are used to simulate small and large leakages, respectively.

**Figure 9:**
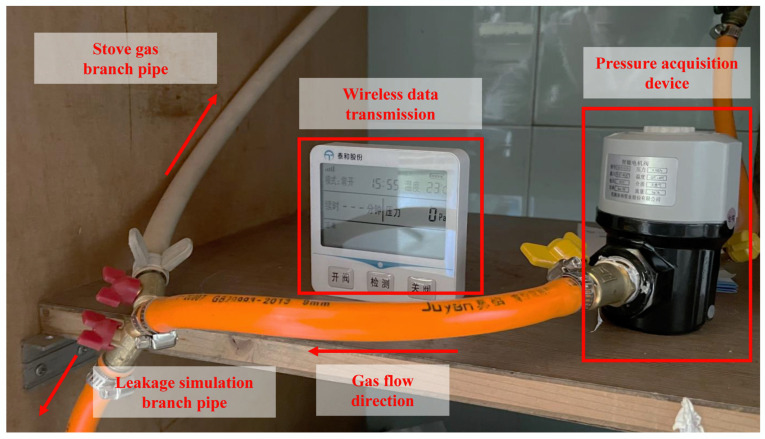
Installation diagram of the leakage simulation branch and pressure monitoring device.

The type of the pressure monitoring device is the TH-GSI, with a range of 0–10 kPa, an accuracy level of 0.1%FS, a supply voltage of 24 VDC, and a 10 Hz acquisition frequency. Figure 9 shows the schematic diagram of the pipeline branch and the pressure acquisition device. The actual signals we collected include the gas usage conditions and leakage conditions. The gas usage conditions include 50 groups of small fire signals and 50 groups of large fire signals. The leakage conditions include 50 groups of small leakage signals and 50 groups of large leakage signals.

### 5.2. Actual Signal Denoising Based on Robust Kalman Filter

Based on the analysis in Section 4.2, the robust Kalman filter has obvious advantages in complex pressure signal denoising. We used the robust Kalman filter to denoise four kinds of actual signals. The ratio of the systematic error to observation error of the filter was set to one thousandth, which basically meets the needs of the actual signal noise reduction. The starting points of the four signals were set as the lowest pressure points within 6 S after the pressure drop occurred. In addition, all of the signals needed to be normalized.

**Figure 10:**
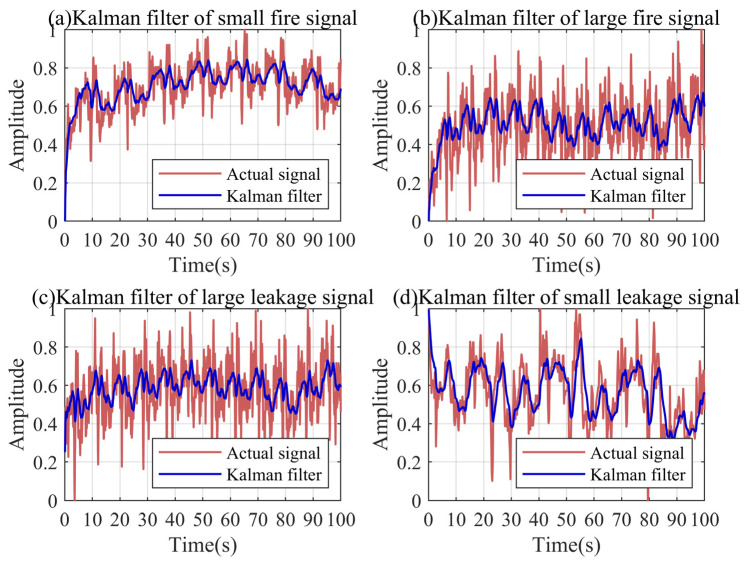
Denoising results of the robust Kalman filter based on the actual signals.

Figure 10 shows the waveforms of four actual signals and the waveforms after the denoising. The actual signals are full of noise, which makes it difficult to judge the periodicity and trend. Through the robust Kalman filter, the characteristics of the signals are clearly displayed. It can be found that the periodic noise structure caused by the membrane meter is similar and the period is different. The small fire signal, large fire signal, and large leakage signal all show an overall increasing trend, while small leakage signal shows an overall decreasing trend. This is because the flow of the small leakage is too small to create a full pressure drop within 6 s. The pressure drop of the other three signals is instantaneous. The pressure fluctuation trend after the pressure drop can identify the leakage. Therefore, the trend features of the different signals need to be further accurately extracted by VMD.

### 5.3. Actual Signal Period Extraction Based on WAA

**Figure 11:**
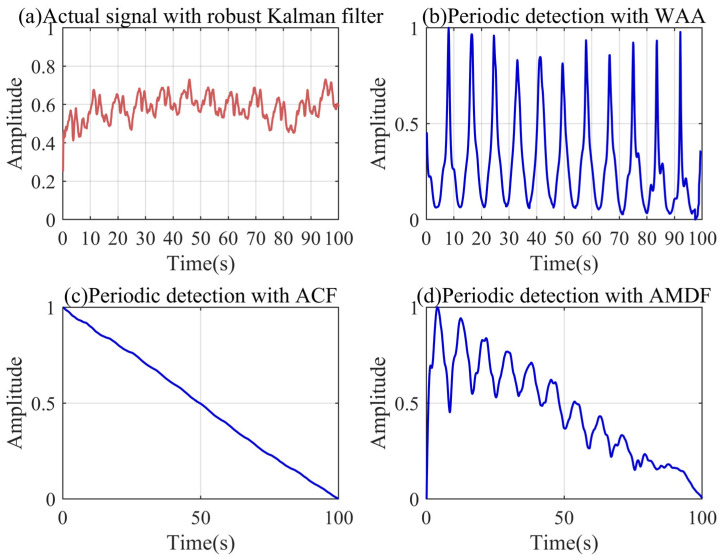
Period extraction results based on the large leakage signal.

In order to accurately calculate the threshold value of the discrete scale-space curve, we used the WAA to extract the period of the actual signal. Figure 11a is the large leakage signal after the robust Kalman filtering for the period extraction. Figure 11b–d show the period extraction results of the WAA, ACF, and AMDF. For complex actual pressure signal sequences, it is almost difficult for the ACF to extract the effective period information. The peak amplitude of the AMDF is significant in a relatively short period of time, but a downward trend is inevitable. On the contrary, the peak structure of the WAA is still significant, and the peak amplitude is basically stable. As analyzed in Section 4.3, the peak structure of the actual periodic noise is more prominent, and the advantage of the WAA is more significant.

### 5.4. Adaptive Spectrum Partition Based on Discrete Scale Space

**Figure 12:**
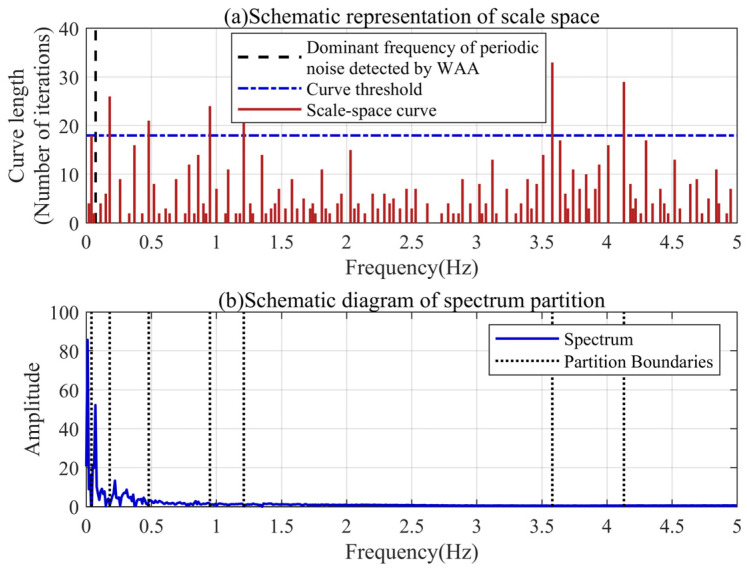
Spectrum partition results based on the small fire signal.

We performed scale-space characterization and the threshold calculation of the filtered spectrum of the real signal using the aforementioned method. Figure 12 shows the scale-space features and frequency band partitioning results of the small fire signal. As shown in Figure 12a, there are more local minima in the actual signal spectrum than in the simulation signal spectrum, illustrating the more complex frequency distribution of the actual signal. Similarly, there are multiple scale-space curves in the region below the dominant frequency of the periodic noise. There are seven curves not lower than the threshold, meaning that the actual signal needs to be decomposed into at least eight IMFs to achieve the separation of the baseline trend.

### 5.5. Improved VMD Decomposition Results of Actual Signal

**Figure 13:**
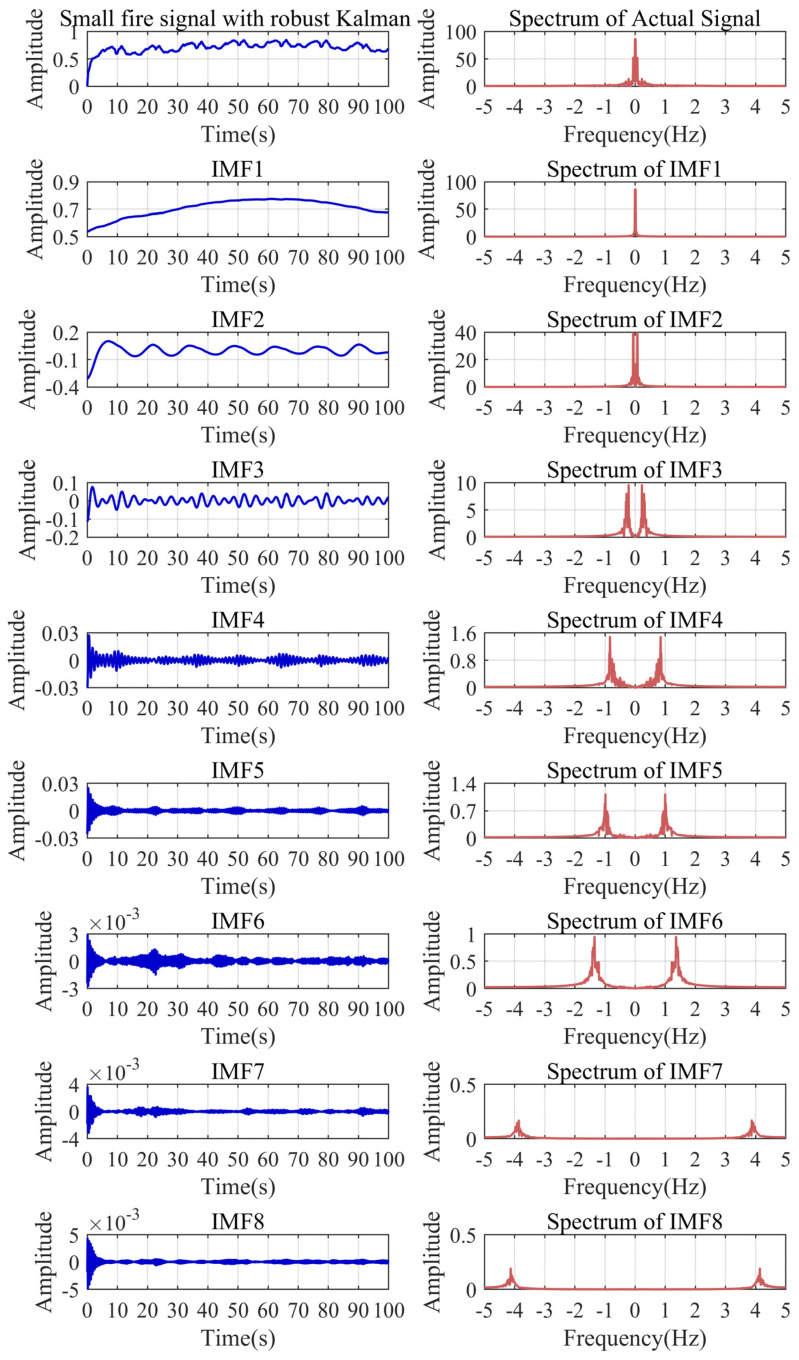
VMD decomposition results of the small fire signal.

**Figure 14:**
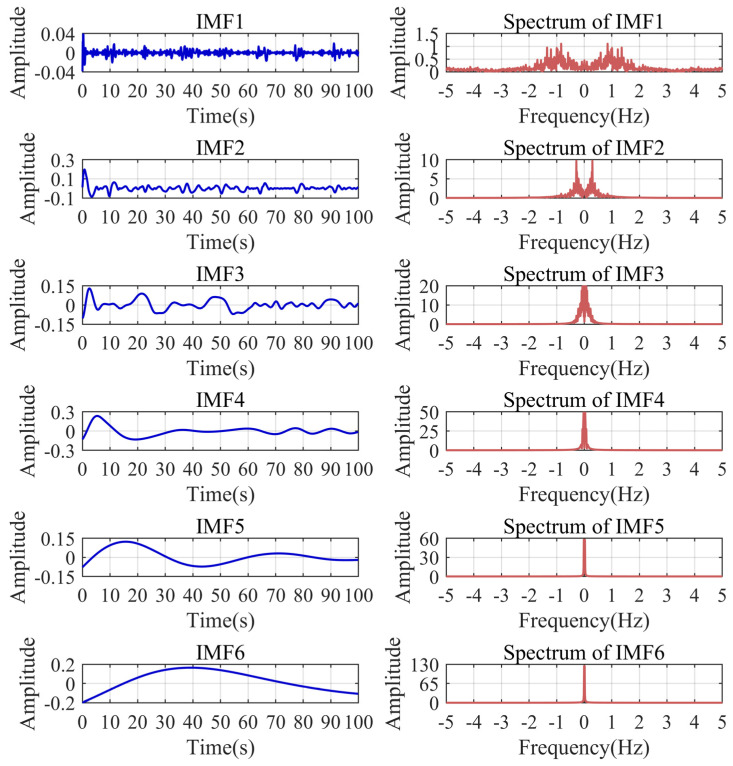
EMD decomposition results of small fire signal.

**Figure 15:**
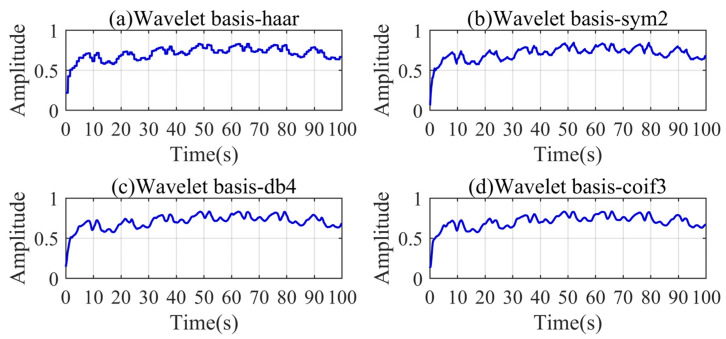
Wavelet decomposition results of the small fire signal.

To illustrate the superiority of the improved VMD, we compared the decomposition results of VMD, EMD, and wavelet analysis on the small fire signal. As shown in Figure 13, the modal parameter *K* and the penalty parameter α of VMD can be set to eight and 7998 through the aforementioned calculation. The wavelet analysis uses four classical wavelet bases for the five-level decomposition. Through Figure 13, Figure 14 and Figure 15, it can be found that only the improved VMD successfully separates the trend baseline and noise.

Compared with the simulated signal, the frequency components in the actual signal are more complicated. EMD has the ability of modal decomposition, but does not have the ability to resist modal aliasing. The frequencies of the IMFs have serious crossover phenomena. Each IMF contains a portion of the low-frequency signal, which is undesirable. Wavelet analysis can filter high-frequency noise, but it is difficult to decompose low-frequency signals. The frequency boundaries of each IMF of VMD are relatively clear. There is a certain amount of aliasing among the three frequency bands of the IMF3, IMF4, and IMF5, resulting in a certain deviation between the actual boundary and the preset boundary. Due to the complex characteristics of the actual signal, mild aliasing cannot be avoided, but it can be confirmed that the frequency bands before and after the preset boundary are effectively separated. Overall, the anti-modal aliasing ability and the effective frequency band separation ability of VMD are outstanding. Some of the IMFs of VMD contain trend information in the initial segment, which can be dealt with by reconstructing the signal.

### 5.6. Reconstruction Results of Actual Signals Based on Fourier Series

**Figure 16:**
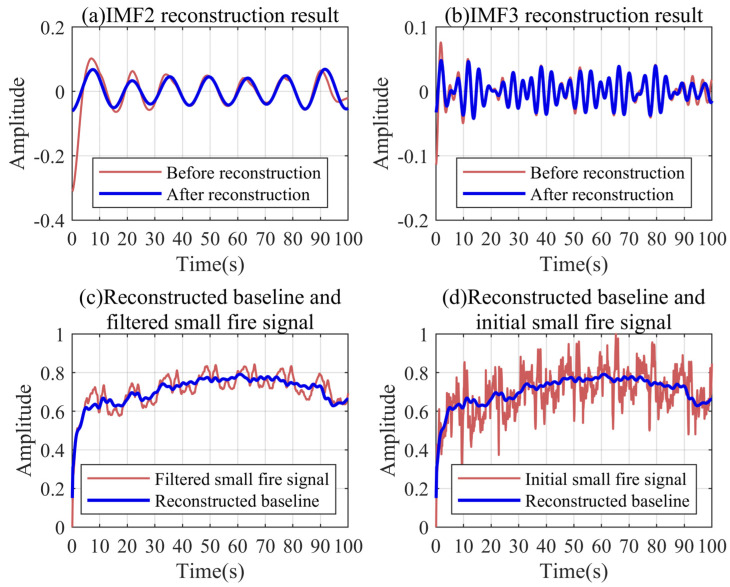
Baseline trend reconstruction results for the small fire conditions.

The low-frequency modal functions obtained by the VMD decomposition need to be reconstructed to obtain the final baseline signal. The low-frequency noise of the small fire signal mainly includes IMF2 and IMF3. Figure 16a,b show the reconstruction results of the adaptive Fourier series for two types of noise. It can be found that the reconstructed signals almost eliminate the trend component caused by the boundary effect. Figure 16c shows the baseline obtained by decomposing and reconstructing the small fire signal after the robust Kalman filtering. The reconstructed baseline basically represents the trend of the small fire signal and completely retains the trend characteristics of the initial part. It can be found from Figure 16d that the reconstructed baseline removes most of the noise. The trend characteristics of the complex pressure signals are clearly displayed.

**Figure 17:**
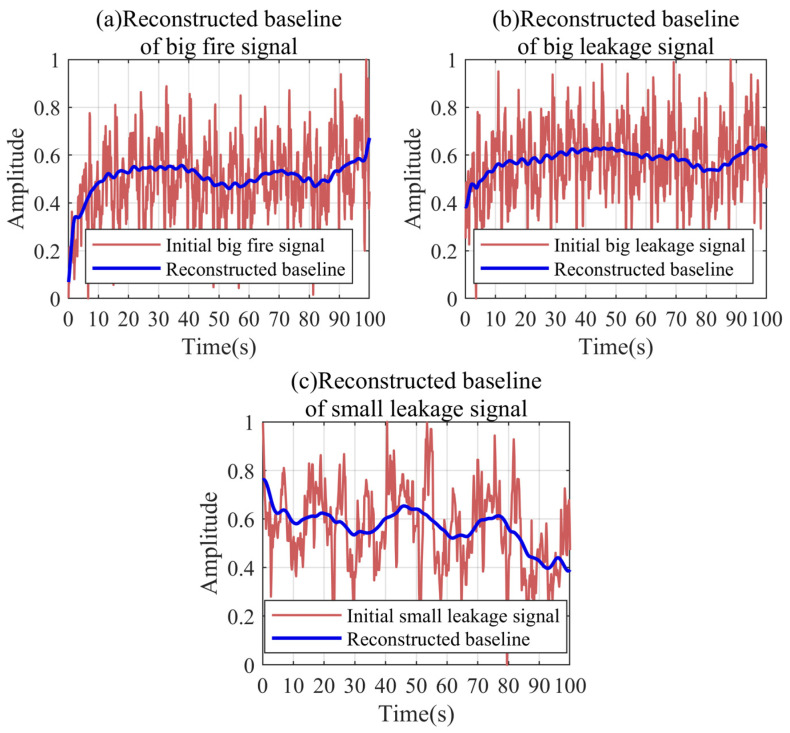
Baseline trend reconstruction results for the different conditions.

Figure 17 shows the reconstruction results of the baseline trends for the large fire signal, the large leakage signal, and the small leakage signal. It can be intuitively found that there are obvious differences in the fluctuation trends of the leakage and gas usage. The pressure fluctuations under the gas usage condition have the characteristics of an obvious increase. The trend of the pressure fluctuation under the leakage condition is almost not increasing, and even decreasing. By reconstructing the baseline, the fluctuation difference between the different conditions can be accurately quantified, providing an identification basis for the leak detection.

### 5.7. Leak Detection Results Based on Actual Signals

Ref. [3] established a negative pressure wave characteristic indicator system for low-pressure pipelines to distinguish gas usage and leakage conditions. The indicators can be input into the extreme learning machine to complete the leak detection. The main basis for the leak detection is the trend of pressure fluctuations within a certain period of time after the pressure drop. In the actual pipeline, the pressure will gradually stabilize within 20 s after it starts to drop. Therefore, we mark the first stage from the inflection point of the pressure drop to the lowest point of the pressure drop, the second stage from the lowest point of the pressure drop to 10 s after the pressure starts to drop, and the third stage from the lowest point of the pressure drop to 20 s after the pressure starts to drop. The mean, variance, linear fitting slope, duration, and head-to-tail difference in the three stages should be calculated, respectively. More details can be found in [3].

In this paper, the complex pressure signals in the low-pressure pipelines were reconstructed into pure negative pressure waveforms. The lowest point of the pressure drop is the starting point of our reconstruction baseline. As mentioned in Section 1, the time-domain waveform of the pressure drop (stage 1) resembles a straight line with a slope close to 90 degrees. Therefore, the calculation of the indicators of the first stage is very simple. The main difference between different conditions is reflected in the oscillation fluctuation trend after the pressure drop. The indicators of the second stage and the third stage calculations based on the reconstructed baseline are the core of the leak detection. Table 1 shows the typical indicators of second stage of the different conditions. It can be found that the trend difference in the different conditions can be clearly displayed by the numerical difference in the indicators.

We used 40 groups of 50 groups of signals for each condition for the training of the extreme learning machine, and 10 sets for the test validation. The screening of the test data is random. We trained and tested three times separately. In order to ensure the reliability of the test, the average of three groups of leak detection accuracy rates was used as the final result. The detection accuracy of the small leakage reached 96.7% and the detection accuracy of the large leakage reached 73.3%. This is mainly due to the obvious difference between the trend characteristics of the small leakage signals and the gas usage signals. In fact, there was only one mistake in the detection of 30 small leakages, which was to judge it as a large leakage. The trend characteristics of the large leakage signals are similar to those of the gas usage signals, both rising for a period of time. The main difference is that the large leakage signal tends to rise more slowly.

## 6. Conclusions

When a negative pressure wave is generated in the low-pressure gas pipeline inside of the building, the fluctuation trend of the pressure is always masked by the noise. Through the robust Kalman filtering and the improved VMD, we established a complete set of pressure fluctuation trend extraction methods. Based on the reconstructed baseline signal, the characteristics of the negative pressure wave under different conditions can be accurately quantified to realize leakage detection. According to the specific signal acquisition environment and typical equipment parameters, the main conclusions are drawn as follows:

(1) Compared with the Kalman filtering, median filtering, and wavelet soft threshold filtering, the robust Kalman filtering has obvious advantages in denoising the complex pressure signals of low-pressure pipelines. It can more accurately estimate the true state of the signal, and has a stronger anti-interference ability with respect to gross errors.
(2) The discrete scale space based on the WAA can reasonably segment the spectrum of the pressure signal, so as to adaptively calculate the modal parameter *K* of VMD. Based on the dominant frequency of the low-frequency periodic noise calculated by the WAA, the calculation model of the penalty parameter α was established.
(3) The improved VMD can effectively separate the baseline signal and noise signal in the complex pressure signal. Compared with EMD and wavelet analysis, the anti-mode aliasing ability and effective frequency band separation ability of the improved VMD are prominent.
(4) The signal adaptive reconstruction method based on the Fourier series can effectively remove the trend component contained in the low-frequency IMF, which is caused by the boundary effect in the VMD decomposition process.
(5) Based on the robust Kalman filter and the improved VMD, the fluctuation trend of the negative pressure wave after the pressure drop can be accurately quantified as the core basis of leakage detection. Based on the reconstructed baseline signal, the detection accuracy of the small leakage reached 96.7% and the detection accuracy of the large leakage reached 73.3%.

In the field of the leakage detection of low-pressure gas pipelines in buildings, the existing method is mainly pressure threshold detection. Our proposed method requires the real-time monitoring of the pressure signal, and taps into the pressure fluctuation characteristics by estimating the pressure wave trend so as to discriminate between the leakage and firing action. The application effect of the proposed method depends on the frequency and accuracy of the pressure monitoring. Therefore, the future research direction focuses on the design of IoT pressure monitoring valves, the construction of a million-level user database, and the development of edge processors. The promotion of large-scale devices and low-cost sensing devices are the main challenges for the application. Since the proposed method relies only on the acquisition of high-quality pressure signals, it does not cause any damage to the environment or safety. It should be noted that the IoT pressure monitoring valve developed based on the proposed method can help the government and gas companies to remotely monitor and disconnect the gas systems scattered inside of buildings, which greatly improves the level of safety management.

## Figures and Tables

**Table 1:** The typical indicators of the second stage of the different conditions.

Characteristic Indicators	Large Fire	Small Fire	Large Leakage	Small Leakage
Mean	2069.5 Pa	2090.6 Pa	2048.3 Pa	2220.8 Pa
Variance	456.3 Pa	724.3 Pa	76.3 Pa	40.3 Pa
Duration	8.5 s	8.2 s	9.1 s	4 s
Linear fitting slope	6.9 Pa/s	12.2 Pa/s	1.4 Pa/s	−3.8 Pa/s
Head-to-tail difference	78 Pa	115.5 Pa	14 Pa	−12 Pa

## Data Availability

Data are contained within the article.
